# Effects of Warm Needle Acupuncture on Temporomandibular Joint Disorders: A Systematic Review and Meta-Analysis of Randomized Controlled Trials

**DOI:** 10.1155/2021/6868625

**Published:** 2021-11-27

**Authors:** Gao-Feng Liu, Zhen Gao, Zheng-Nan Liu, Min Yang, Sheng Zhang, Tai-Peng Tan

**Affiliations:** ^1^Graduate Faculty, Tianjin University of Traditional Chinese Medicine, Tianjin 301617, China; ^2^Acupuncture and Tuina School/The 3rd Teaching Hospital, Chengdu University of Traditional Chinese Medicine, Chengdu 6100752, China; ^3^The First Affiliated Hospital of Guangzhou University of Traditional Chinese Medicine, Guangzhou 510405, China; ^4^Dermatology Department, Qujiang District Hospital of Traditional Chinese Medicine, Quzhou 324022, China; ^5^Neurology Department, Qujiang District Hospital of Traditional Chinese Medicine, Quzhou 324022, China; ^6^Acupuncture Department, Heilongjiang Provincial Academy of Traditional Chinese Medicine Sciences, Harbin 150036, China

## Abstract

**Background:**

Temporomandibular joint disorders (TMDs) are a common and prevalent disease with main symptoms of pain, joint sounds, and mandibular movement disorders, which seriously affects the mental health and quality of life of the sufferers. In recent years, there have been an increasing number of studies utilizing warm needle acupuncture (WNA) for the treatment of TMD, and the quality of the studies has gradually improved. However, evidence from evidence-based medicine is lacking. This study aims to use a systematic review and meta-analysis method to understand the efficacy of WNA for the treatment of TMD. *Methods and Analysis.* We searched randomized controlled trials (RCTs) of WNA for the treatment of TMD from 9 electronic databases, including 5 English databases (PubMed, EMBASE, Cochrane Library, Web of Science, and MEDLINE) and 4 Chinese databases (Chinese National Knowledge Infrastructure (CNKI), Chinese VIP Information, Wanfang Database, and Chinese Biomedical Literature Database (CBM)) from their inception to May 2021. The included RCTs compared WNA with acupuncture, electroacupuncture, pharmacological therapy, or other therapies. And outcome indicators such as total effective rate and cure rate were assessed. All analyses were conducted using RevMan software V5.3 and Stata16. Measurement count data used the relative risk (RR) as the efficacy statistic, and each effect size was given its point estimate value and 95% confidence interval (CI).

**Results:**

The meta-analysis included 10 studies with a total of 670 patients, which included 340 patients in the experimental group and 330 patients in the control group. The data in this review showed that WNA is superior to treatments such as acupuncture alone, acupuncture therapy combined with TDP, drug therapy, and ultrasonic therapy in terms of effective rate (RR = 1.20; 95% CI, 1.06 to 1.35; and *P* = 0.003) and cure rate (RR = 1.82; 95% CI, 1.46 to 2.28; and *P* < 0.00001) for the treatment of TMD.

**Conclusions:**

This systematic review and meta-analysis provides new evidence for the effectiveness of WNA for the treatment of TMD. However, the above conclusions need to be further verified by multicenter prospective studies of larger samples and higher-quality RCTs. Protocol registration number: INPLASY202160030.

## 1. Introduction

Temporomandibular joint disorder (TMD) is a general term for a series of diseases involving the temporomandibular joint, masticatory muscles, and surrounding structures. Mouth-opening limitation, oral and maxillofacial pain, and joint clanging are the most common symptoms, which severely affects the quality of life of patients with TMD [1, 2]. Anxiety, depression, somatization, and other psychological factors are closely related to the generation of TMD [3, 4]. Epidemiological study has shown that about 10% of the population is affected by TMD, especially in 30-year-old women [5]. Because the etiology of TMD is not clear, TMD patients mainly come to see the doctor with pain and abnormal bite. Clinically, there are abundant treatment methods with different efficacies, such as drug treatment, psychological treatment, occlusal treatment, physical therapy, acupuncture treatment, fire needle treatment, and surgical operation [6]. Warm needle acupuncture (WNA) is a commonly used method to treat TMD, which combines the advantages of acupuncture and moxibustion. The needle is pierced to an appropriate depth, and then the moxa on the handle of the needle is heated, so that the warm stimulus will transmit to the deep part of the acupuncture point through the needle, which helps to dispel wind and cold to relieve pain. In recent years, clinical research on WNA for the treatment of TMD has gradually increased. However, there is still insufficient evidence to support the effectiveness of WNA in the treatment of TMD. Therefore, we performed a systematic review and meta-analysis (participants: patients with TMD; interventions: WNA; comparisons: acupuncture, electroacupuncture, pharmacological therapy, or other therapies; outcomes: total effective rate and cure rate; and study design: randomized controlled trials) to evaluate the effectiveness of WNA in the treatment of TMD and to provide references for the clinical research and application of WNA to treat TMD.

## 2. Methods

### 2.1. Study Registration

This protocol was registered in the International Platform of Registered Systematic Review and Meta-analysis Protocols. The register name is “Effects of warm needle acupuncture on temporomandibular joint disorders: a systematic review and meta-analysis of randomized controlled trials.” The registration number was INPLASY202160030. In addition, we conducted this meta-analysis according to the Preferred Reporting Items for Systematic Reviews and Meta-Analysis (PRISMA) guideline [7].

### 2.2. Ethics

Since this systematic review is based on published research, the approval of the ethics committee is not required.

### 2.3. Eligibility Criteria

#### 2.3.1. Types of Study

We had searched all randomized controlled trials (RCTs) on WNA for the treatment of TMD. The language was restricted to Chinese and English.

#### 2.3.2. Object of Study

Patients are clearly diagnosed with TMD (age, gender, course of disease, and other factors are not limited).

#### 2.3.3. Intervention

The test group was treated only with WNA intervention, while the control group adopted acupuncture, electroacupuncture, pharmacological therapy, or some other means of treatment.

#### 2.3.4. Outcome Indicators

Total effective rate and cure rate.

### 2.4. Search Methods for Identification of Study

We had searched the following electronic databases: PubMed, EMBASE, Cochrane Library, Web of Science, MEDLINE, CNKI, Chinese VIP Information, Wanfang Database, and CBM, from their inception to May 2021 to retrieve all RCTs related to WNA for the treatment of TMD (the search terms used in Chinese databases had the same English meaning).  Table 1 provides detailed search strategies in PubMed, which were also used in other databases. In addition, we manually searched the references in all located articles to find further related articles.

### 2.5. Study Selection and Data Extraction

Firstly, two reviewers (GFL and ZG) independently screened the retrieved documents by using Endnote to manage the retrieved documents and remove repetitive literature. Then, a preliminary screening was conducted by reading the title and abstract to exclude unrelated studies, especially the studies about animal experiments, expert experience, incomplete articles, review articles, and case reports. A further screening was conducted by reading the full text to remove the research studies that showed mismatched intervention measures, missing data, and nonrandomized controlled trials. Besides, any disagreements would be resolved through discussions with the third reviewer (MY).

**Figure 1:**
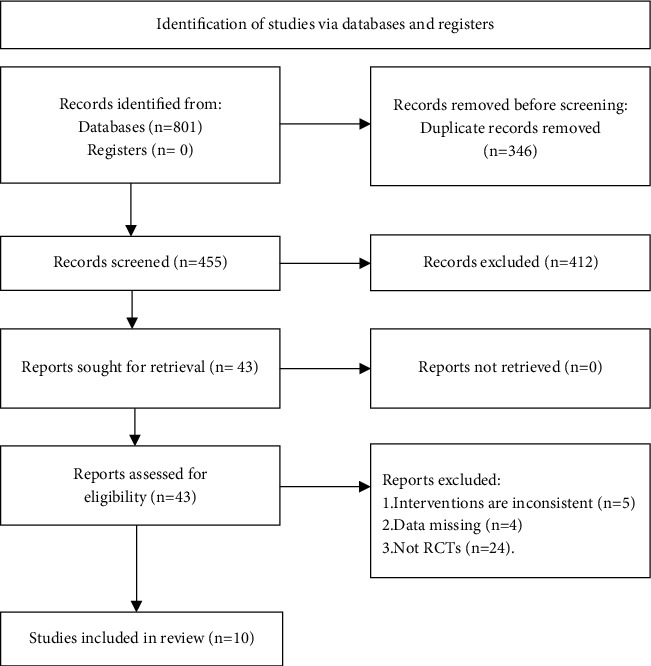
Literature search diagram.

The entire selection process was shown in Figure 1. After selection, the relevant information was extracted from the final included research, including the author, publication year, intervention measures, sample information (size, age, gender, and disease course), treatment process, key elements of bias risk evaluation, outcome indicators, and outcome measurement data (total effective rate and cure rate), and so on.

### 2.6. Assessment of Risk of Bias

The RCT bias risk assessment tool recommended by Cochrane Handbook 5.1.0 was used to evaluate the randomization method, allocation hiding, blinding method, completeness of the result data, selective result reporting, and other sources of bias in the included studies. The evaluation was independently conducted by two researchers, and the results were cross-checked. In addition, the disagreement would be resolved by a discussion [8].

### 2.7. Statistical Analysis

Revman5.3 and Stata16 software was used for statistical analysis of the outcome indicators. Measurement count data used the relative risk (RR) as the efficacy statistic, and each effect size was given its point estimate value and 95% confidence interval (CI). In some studies, there were inconsistencies in the inclusion criteria, course, and severity of the patients. The random effect was used in all the studies, as they are heterogeneous. To explore the sources of heterogeneity, a subgroup analysis was conducted, while sensitivity analysis was used to analyze robustness and reliability. Funnel charts were used to assess potential publication bias, and *P* < 0.05 was considered statistically different.

## 3. Results

### 3.1. Study Selection

A total of 801 documents were obtained through the search, and 455 were obtained after excluding the duplicate documents. After reading the title and abstract, 412 documents were eliminated, and 33 unqualified documents were further eliminated by reading the full text. Finally, 10 documents were qualified and included (see Figure 1) [9–18].

### 3.2. Study Characteristics

A total of 10 [9–18] studies with 670 patients were included, including 340 in the experimental group and 330 in the control group. The WNA was used for intervention in the experimental groups. Five [10, 13, 15, 16, 18] studies in the control group used drug therapy, two [9, 14] studies used acupuncture, and three studies used acupuncture therapy combined with TDP [11], ultrasonic therapy [12], and electroacupuncture [17] treatments, respectively. The basic characteristics of the included studies are shown in Table 2 and 3.

### 3.3. Risk of Bias

**Figure 2:**
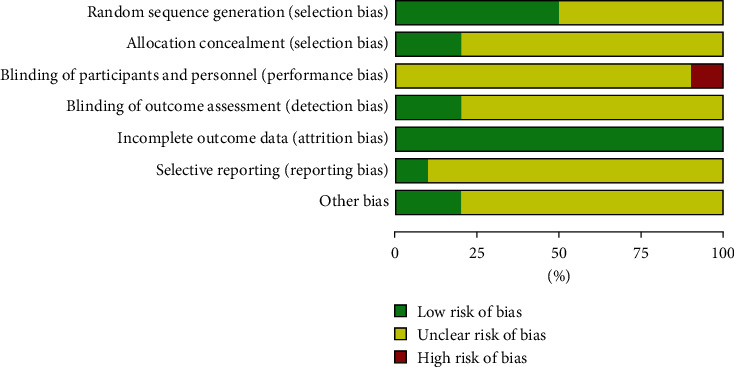
The risk deviation ratio chart of the included studies.

**Figure 3:**
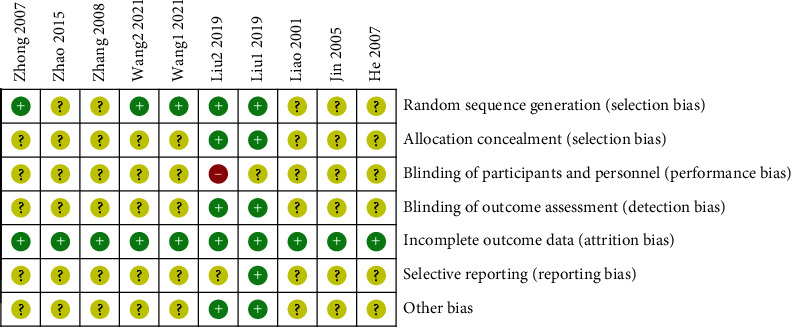
General chart of the methodological quality evaluation of the included studies.

We evaluated the randomization method, allocation hiding, blinding method, completeness of the result data, selective result reporting, and other sources of bias in the included studies according to the Cochrane risk of bias assessment tool for quality evaluation. In terms of random sequence generation, 5 studies ([12], Liu 1 [15], Liu 2 [16], Wang 1 [17], and Wang 2 [18]) used a random number table method and the risk of bias was assessed as low risk, while the other studies did not mention randomization methods. Except for 2 studies (Liu 1 [15] and Liu 2 [16]) showing that allocation concealment had a low risk, no other studies mentioned allocation concealment. One study (Liu 2 [16]) was an open study, and the blinding of the subjects was assessed as high risk, while the blinding status of the remaining subjects was not provided with specific descriptions. Two studies (Liu 1 [15] and Liu 2 [16]) mentioned the blinding of the evaluator with no other bias. Other studies did not mention the blinding of the evaluator, and the source of the other biases was not clarified. One study (Liu 1 [15]) had a complete report on the study protocol and results, which was rated as low risk, while other studies were unspecified. The specific risk bias assessment is shown in Figure 2 and 3.

### 3.4. Total Effective Rate

**Figure 4:**
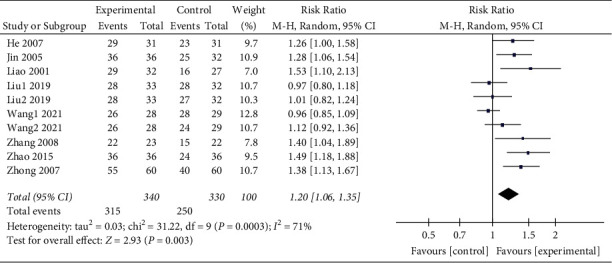
Total effective rate forest map.

The 10 [9–18] included studies all reported the total effective rate. Through the combined analysis of binary categorical variables, the heterogeneity results (*P* = 0.0003 and I^2^ = 71%) indicated that there were heterogeneous among the included 10 studies. Thus, the random effects model analysis was used, and the combined RR value (RR = 1.20 and 95% CI: 1.06 to 1.35) suggested that WNA was better than other methods in treating TMD. The combined statistical test (*Z* = 2.93 and *P* = 0.003) was statistically significant. The total efficient forest map is shown in Figure 4.

**Figure 5:**
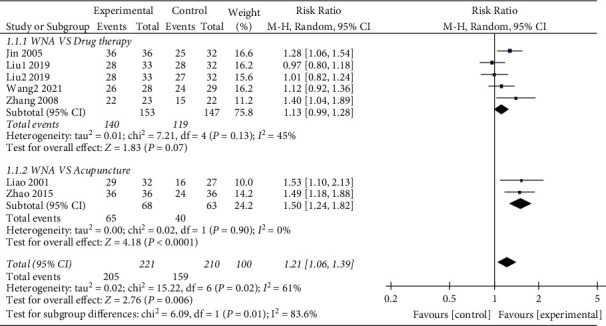
Forest map for total efficiency subgroup analysis.

The subgroup analysis by different categories of control interventions on the total effectiveness rate showed that WNA has a better effect compared with drug therapy (RR = 1.13; 95% CI: 0.99 to 1.28; and *p* = 0.07) and acupuncture (RR = 1.50; 95% CI: 1.24 to 1.82; and *p* < 0.0001) (Figure 5).

### 3.5. Cure Rate

**Figure 6:**
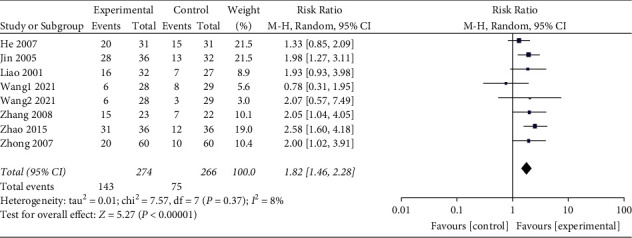
Forest graph of the cure rate.

Eight [9–14, 17, 18] of the included studies reported the cure rate. Through the combined analysis of binary variables, the heterogeneity results showed that the included studies had no heterogeneity (*P* = 0.37 and I^2^ = 8%). The random effects model analysis was used, and the combined RR value (RR = 1.82 and 95% CI: 1.46 to 2.28) combined with a statistical test (*Z* = 5.27, *P* < 0.00001) showed the difference between the two groups was statistically significant, indicating that the cure rate of the test group was higher than that of the control group. The forest graph of cure rate is shown in Figure 6.

### 3.6. Sensitivity Analysis

**Figure 7:**
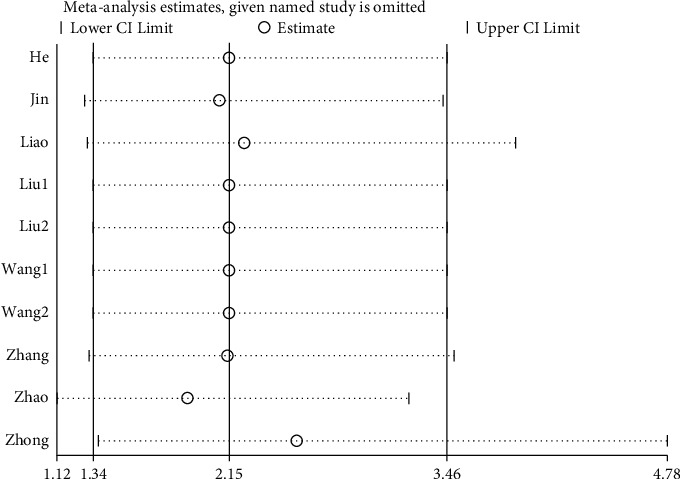
Sensitivity analysis of the total effective rate: the lowest and highest values of the confidence interval were 1.12 and 4.78, respectively; the upper and lower limits of the meta-analysis effect size and the confidence interval were 1.34 and 3.46, respectively.

**Figure 8:**
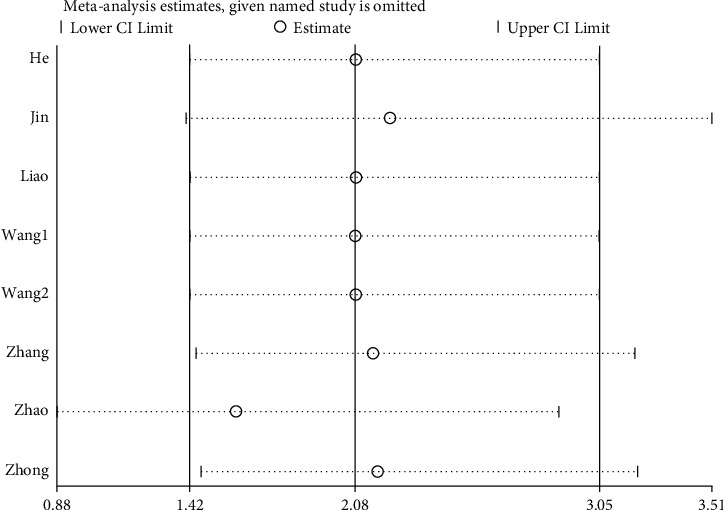
Sensitivity analysis of cure rate: the lowest and highest values of the confidence interval were 0.88 and 3.51, respectively; the upper and lower limits of the meta-analysis effect size and the confidence interval were 1.42 and 3.05, respectively.

Through a sensitivity analysis of the total effective rate and cure rate of WNA for the treatment of TMD, the results showed the same conclusion as the original conclusions, whether it is to compare the results of the included studies one by one with the results before they are not eliminated, or to change the effect model. The sensitivity analysis suggested that this research has good stability, and the results are shown in Figures 7 and 8.

### 3.7. Publication Bias

A funnel chart was drawn for the publication bias of the 10 studies [9–18], which used the total effective rate as an outcome indicator. The results showed that the study points of the funnel chart of each index were symmetrically distributed on the left and right sides. The *P* values of Egger's test and Begg's test were both ≥ 0.05 (Begg's test: *P* = 0.221; Egger's test: *P* = 0.300), both of which indicated no significant publication bias.

A funnel chart was drawn for the publication bias of 8 studies [9–14, 17, 18], which used the cure rate as an outcome indicator. The results showed that the study points of the funnel chart for each index were symmetrically distributed on the left and right sides. The *P* values of both Egger's test and Begg's test were both ≥ 0.05 (Begg's test: *P* = 0.089; Egger's test: *P* = 0.066), suggesting no significant publication bias.

## 4. Discussion

There are many studies in literature of systematic reviews and meta-analyses on TMD, partly focusing on epidemiological studies, partly on complications, and more often on a particular treatment modality. WNA mentioned in this paper is an innovative therapy of traditional Chinese medicine, and no previous meta-analysis has been reported on the treatment of TMD with WNA. This systematic review and meta-analysis included 10 RCTs with a total of 670 patients, including 340 in the experimental group and 330 in the control group. The data in this meta-analysis showed that WNA is superior to acupuncture alone, acupuncture therapy combined with TDP, drug therapy, ultrasonic therapy, and electric acupuncture in terms of effectiveness and cure rate for the treatment of TMD (total effective rate: *Z* = 2.93, *P* = 0.0006; cure rate: *Z* = 5.27, *P* < 0.00001), suggesting that WNA is one of the effective methods for the treatment of TMD.

TMD is a common and frequently occurring disease. The overall prevalence of temporomandibular joint disorders in adults/the elderly and children/adolescents is approximately 31% and 11%, respectively [19]. The incidence of TMD is also related to occupation. The overall combined prevalence of TMD among musicians is about 53.9% [20]. The main symptoms of TMD include muscle and joint-related pain, decreased jaw movement, headache, tinnitus [21], stiffness, fatigue, and other potentially related symptoms [22]. Bruxism would be associated with TMD [23]. Studies reported that 72% of the TMD patients (especially elderly women) were accompanied by ear symptoms. 49% of the TMD patients had ear occlusion as the main manifestation, which was related to oral dysfunction on the ipsilateral side and muscle pain during palpation [24]. The etiology of TMD is complex and is closely related to immune factors, joint anatomical factors, occlusal factors, psychosocial factors (such as anxiety and depression), and physical symptoms [25]. Patients with TMD have a higher prevalence of moderate somatization and depression, while severe physical injury is not common [26]. Psychological and physical ailments caused by TMD result in a lower quality of life in patients [27].

There are many clinical treatment methods for TMD, including traditional Chinese medicine, acupuncture, massage, nonsteroidal anti-inflammatory drugs, partial closure of hormones and local anesthetics, orthodontic training of oral pads, laser, shock wave, clamp compression, manual reduction, and muscle energy [28–30]. Particularly, physical therapy (PT), including mandibular movement, ultrasound, manual therapy (MT), acupuncture, and laser therapy, are considered effective treatment options for TMD [31].

TMD belongs to the categories of “arthralgia,” “cheek pain,” and “jaw pain” in Chinese medicine. Due to the weakness of the body's Qi and blood, wind, cold, and dampness, invade and close and block the face, resulting in poor Qi and blood flow in the face and loss of nourishment for muscles and joints, which cause symptoms such as unfavorable joint opening, popping, and pain [32, 33]. WNA combines acupuncture and moxibustion, which can play the dual role of acupuncture and moxibustion, and the effect is stronger than simple acupuncture [34]. Through its warming effect, warm acupuncture can promote blood circulation, relieve muscle spasms, and improve the functional activities between ligaments, joint capsules, and condyles, and eliminate symptoms. At the same time, the warming effect can inhibit sensory nerves and reduce nerve excitability, which can achieve an analgesic effect [35, 36]. Studies have shown that both acupuncture and moxibustion can have an anti-inflammatory and analgesic effect by regulating the expression of inflammatory factors, inhibiting pain sensitization, and regulating ion function channels [37, 38]. This meta-analysis shows that WNA is effective in improving joint dysfunction and pain caused by TMD and is superior to acupuncture, western medicine, electroacupuncture, TDP, and other treatment methods, providing a scientific basis for further clinical applications.

The previous meta-analysis shows that acupuncture and nonsteroidal anti-inflammatory drugs can effectively reduce the pain of patients with TMD [6, 39]. Low-level laser therapy, occlusal splints therapy, and exercise therapy can effectively improve mandibular movement and relieve pain in patients with TMD [40–42]. These methods have certain clinical effects but are mostly limited to symptomatic treatment, lacking a unified treatment plan and standards, resulting in the limitation of clinical evidence. In particular, long-term follow-up is required to determine the long-term relative efficacy and safety of these treatments. This study also had some limitations: (1) this study only retrieved Chinese and English literature, and there may be incomplete retrievals. (2) In some studies, there were inconsistencies in the inclusion criteria, course, and severity of the patients. (3) The included studies mostly were single-center studies and the randomized control sample size was small, which may lead to admission bias and selection bias due to a lack of multicenter and large-sample collaborative research. Therefore, a large sample, multicenter, and higher-quality clinical trial will still be needed for further verification. (4) The included studies lack reports on side effects. (5) There were two studies (Liu [15, 16] and Wang [17, 18]) that showed that the population was practically the same, but the results were published separately. This had an impact on the conclusions.

## 5. Conclusions

The results of the systematic review and meta-analysis showed WNA may have had a significant therapeutic effect and clinical significance for TMD when compared with acupuncture, acupuncture therapy combined with TDP, drug therapy, ultrasonic therapy, and electric acupuncture. The poor quality of primary studies hinders the possibility of any real conclusion about the intervention. So, meta-analyses do not offer a reliable result regarding the real effectiveness of the WNA on TMD. The low quality of the original study was due to the small number of people currently conducting this study. Therefore, more high-quality and large-sample collaborative prospective studies are needed to further confirm the efficacy of WNA on TMD. Although further research is needed to determine the effectiveness of WNA in the treatment of TMD, WNA may be one of the effective methods to treat TMD in clinic, and we hope that the publication of this meta-analysis could promote the study of WNA in the treatment of TMD.

## Figures and Tables

**Table 1:** Search strategy in PubMed.

NO.	Search details	Results
#1	“Temporomandibular joint Disorders”[MeSH terms]	17827
#2	(((“Joint s” [All fields] OR “joints” [MeSH terms] OR “joints” [All fields] OR “Joint” [All fields]) AND “disorder *∗* “[All fields]) AND “Temporomandibular” [Title/Abstract]) OR (((“joint s”[All fields] OR “joints” [MeSH terms] OR “joints”[All fields] OR “Joint”[All fields]) AND “disease*∗*“[All fields]) AND “Temporomandibular” [Title/Abstract]) OR (“disorder *∗*“ [All fields] AND “Temporomandibular” [Title/Abstract]) OR (“disease *∗* “[All fields] AND “temporomandibular joint” [Title/Abstract]) OR “temporomandibular disorder *∗*“ [Title/Abstract] OR “temporomandibular joint disease”[Title/Abstract] OR “TMJ” [Title/Abstract]	18806
#3	#1 AND #2	12593
#4	“Acupuncture” [Title/Abstract] OR “Moxibustion” [Title/Abstract] OR “wen zhen” [Title/Abstract] OR “needle warming moxibustion” [Title/Abstract] OR “needle warming therapy” [Title/Abstract] OR “warm needling” [Title/Abstract] OR “warm acupuncture” [Title/Abstract] OR “warm needle moxibustion” [Title/Abstract] OR (“Warm” [All fields] AND “acupuncture-moxibustion”[Title/Abstract]) OR “warm needle acupuncture” [Title/Abstract] OR (“warming acupuncture” [Title/Abstract] AND “Moxibustion” [Title/Abstract])	25076
#5	#3 AND #4	138

**Table 2:** Characteristics information of the included studies.

Author	Year	Group	Interventions	Sample size	Age (years)	Gender (M/F)
Liao	2001	T	WNA	32	15–52	21/38
		C	Acupuncture	27		
Jin	2005	T	WNA	36	19–61	10/26
		C	Drug therapy	32	21–59	8/24
He	2007	T	WNA	31	20.48 ± 5.30	NA
		C	Acupuncture + TDP	31	18.24 ± 5.21	
Zhong	2007	T	WNA	60	35 ± 5	30/30
		C	Ultrasonic therapy	60	34 ± 4	30/30
Zhang	2008	T	WNA	23	18–55	13/10
		C	Drug therapy	22	18–55	10/12
Zhao	2015	T	WNA	36	18–45	15/21
		C	Acupuncture	36	19–50	18/18
Liu 1	2019	T	WNA	33	48 ± 0	14/19
		C	Drug therapy	32	49 ± 1	15/17
Liu 2	2019	T	WNA	33	48.42 ± 0.28	14/19
		C	Drug therapy	32	48.91 ± 0.67	15/17
Wang 1	2021	T	WNA	28	44.14 ± 14.16	4/24
		C	Electroacupuncture	29	39.24 ± 12.97	5/24
Wang 2	2021	T	WNA	28	44.14 ± 14.16	4/24
		C	Drug therapy	29	43.91 ± 16.34	6/23

Note. *T* = test group, *C* = control group, I = intervention, *M* = male, *F* = female.

**Table 3:** Characteristics information of the included studies.

Author	Year	Course of the disease	Course of treatment	Outcome indicators
Liao	2001	7 days–2 years	Once a day, 10 times a course of treatment, 2 courses of treatment.	①②
Jin	2005	1 weeks–8 months	Once a day for 5 days, 1 course of treatment, 1 day interval, consecutive 2 courses of treatment.	①②
		1 weeks–6 months	Diazepam 2.5 mg and indomethacin 25 mg were taken orally, 3 times a day, one course for 5 days, 2 courses.	
He	2007	11.33 ± 4.52 days	Once a day, 10 times a course of treatment, 2 courses of treatment, and the interval of courses is 2–3 days.	①②
		10.44 ± 3.88 days		
Zhong	2007		Once a day for 10 consecutive times.	①②
Zhang	2008	10 days–6 months	Once a day, 10 times a course of treatment, 2 courses of treatment.	①②
			Oral indomethacin 25 mg, 3 times a day and vitamin B1 10 mg, 3 times a day. Mental tension diazepam tablets 2.5 mg, once a day, taken before going to bed. One course of treatment for 10 days, 2 courses.	
Zhao	2015	Less than 6 years	Once a day, 7 times a course of treatment, an interval of 2 days, 2 courses of treatment.	①②
Liu 1	2019	3.98 ± 1.96 months	The treatment was performed once every other day, 3 times a week for 4 weeks.	①②
		4.27 ± 1.84 months	Oral diclofenac sodium sustained-release capsule (Zhejiang Sincerity Pharmaceutical Co., Ltd.,) (75 mg per granule) twice a day; oral glucosamine hydrochloride tablets (Yongxin Pharmaceutical, 0.24 g per tablet), 0.48 g each time, 3 times a day. Take both drugs for four weeks.	
Liu 2	2019	3.98 ± 1.96 months	Once every other day, 3 times a week, a total treatment for 4 weeks.	①
		4.27 ± 1.84 months	Oral diclofenac sodium sustained-release capsule (Zhejiang Sincerity Pharmaceutical Co., Ltd.,) (specifications: 75 mg/pill), 1 pill/time, 2 times/d. Glucosamine hydrochloride capsules (Hong Kong Aomei pharmaceutical factory) (specifications: 0.75 g/granule), 1 granule/time, 2 times/d. Both drugs were administered for 4 weeks.	①
Wang 1	2021	10.00 ± 8.87 days	Once a day, 5 times a course of treatment, with a treatment interval of 2 days, for a total of 2 courses.	①②
		8.48 ± 5.88 days		
Wang 2	2021	10.00 ± 8.87 days	Once a day, 5 times a course of treatment, with a treatment interval of 2 days, for a total of 2 courses.	①②
		8.28 ± 6.84 days	Voltaren external application is used 3 times a day, 5 times a course, and 2 days rest, for a total of 2 courses.	

Note. *T* = test group, *C* = control group, I = intervention, outcome indicators: ① total effective rate; ② cure rate.

## Supplementary Materials

Supplementary Materials From: Page MJ, McKenzie JE, Bossuyt PM, Boutron I, Hoffmann TC, Mulrow CD, et al. The PRISMA 2020 statement: an updated guideline for reporting systematic reviews. BMJ 2021; 372: n71. doi: 10.1136/bmj.n71.
